# Management of Erectile Dysfunction in Middle-Aged Men: A Prospective Evaluation of Lifestyle and Pharmacological Treatments in a Tertiary Care Center

**DOI:** 10.7759/cureus.102020

**Published:** 2026-01-21

**Authors:** Santosh Bokre, Deepak Jumani, Anil Waghmare, Satish P Dipankar

**Affiliations:** 1 Physiology, All India Institute of Medical Sciences, Mangalagiri, Mangalagiri, IND; 2 Medicine, Grant Medical College and Sir Jamsetjee Jeejebhoy (JJ) Group of Hospitals, Mumbai, IND; 3 Physiology, Byramjee Jeejeebhoy (BJ) Government Medical College, Pune, IND

**Keywords:** erectile dysfunction, lifestyle modification, pharmacological intervention, quality of life, sexual health

## Abstract

Background

Erectile dysfunction (ED) is a common condition in middle-aged men and is frequently associated with metabolic and cardiovascular comorbidities. Pharmacological therapy is effective for symptom control, but lifestyle modification may provide additional benefits when used in combination.

Objective

The objective of this study is to evaluate changes in erectile function and selected metabolic and hormonal parameters following combined lifestyle modification and pharmacological therapy in middle-aged men with erectile dysfunction.

Methods

This prospective observational study enrolled 60 men aged 40-65 years with erectile dysfunction and at least one metabolic or cardiovascular comorbidity. All participants received daily tadalafil (5 mg), along with structured counselling on diet, physical activity, sleep hygiene, weight management, smoking cessation, and alcohol reduction for three months. Erectile function was assessed using the Sexual Health Inventory for Men (SHIM) questionnaire. Laboratory parameters, including glycated hemoglobin (HbA1c), lipid profile, serum testosterone, and body weight, were measured at baseline and at three months. Paired t-tests were used to compare pre-intervention to post-intervention values, with statistical significance set at p < 0.05.

Results

The mean SHIM score increased from 14 ± 4 at baseline to 22 ± 5 at three months (mean change: +8 points; 95% CI: 6.2-9.7; p < 0.05). HbA1c decreased from 7.9% ± 1.5% to 6.8% ± 0.8% (-13.9%; p < 0.05), and LDL cholesterol decreased from 200 ± 50 mg/dL to 75 ± 20 mg/dL (-62.5%; p < 0.05). Mean total testosterone levels increased from 270 ± 60 ng/dL to 500 ± 120 ng/dL (+85.2%; p < 0.05), while mean body weight decreased from 85.0 ± 12 kg to 79.0 ± 9 kg (-7.1%; p < 0.05). No serious adverse events were reported.

Conclusion

In this prospective observational study, combined lifestyle modification and pharmacological management were associated with improvements in erectile function, glycemic control, lipid profile, serum testosterone levels, and body weight among middle-aged men with erectile dysfunction and cardiometabolic comorbidities. Further controlled studies are needed to confirm these findings and assess long-term outcomes.

## Introduction

Erectile dysfunction (ED) is defined as a persistent inability to achieve or maintain an erection sufficient for satisfactory sexual performance. It has multiple causes and may involve vascular, neurological, hormonal, or psychological factors [1]. Globally, ED is increasing in prevalence, with projections estimating over 322 million affected men by 2025 [2]. Studies such as the Massachusetts Male Aging Study report that complete ED is three times more common in men aged 70 than in those aged 40, highlighting its significant impact on middle-aged men [3].

ED is strongly associated with diabetes, hypertension, dyslipidemia, and obesity, all of which can impair vascular function and reduce nitric oxide (NO) levels [4]. These conditions promote chronic inflammation and oxidative stress, decreasing penile blood flow and complicating erectile function. Men with type 2 diabetes or obesity frequently exhibit low testosterone levels [5]. The rising prevalence of diabetes contributes to long-term vascular and neurological complications, including sexual dysfunction in both sexes. ED is the most common sexual disorder among men with diabetes, who are approximately 3.5 times more likely to experience ED than men without diabetes [6].

The primary treatment for ED involves phosphodiesterase type 5 (PDE-5) inhibitors. Although effective, these medications do not address underlying health conditions or lifestyle factors [7]. Recent evidence indicates that regular physical activity, a plant-based diet, weight reduction, and improved sleep can significantly enhance vascular health and erectile function [8]. Therefore, combining lifestyle interventions with medication may provide more durable benefits [6].

This study evaluated the combined effect of lifestyle modifications and medication on erectile function in middle-aged men with comorbidities at a tertiary care hospital. The primary focus was on changes in erectile function scores, metabolic health, and hormone levels following a three-month intervention.

## Materials and methods

Study design

This prospective observational research study was conducted at the Sexual Health Clinic of Grant Medical College and Sir Jamsetjee Jeejebhoy (JJ) Group of Hospitals, Mumbai, a tertiary care sexual health clinic in India, between December 2023 and December 2024. The study was designed to evaluate changes in erectile function and selected metabolic and hormonal parameters following the routine, multimodal management of erectile dysfunction (ED) in middle-aged men with associated cardiometabolic comorbidities. As an observational study, no control or comparator group was included.

Ethics statement

The study protocol was approved by the Institutional Ethics Committee of Smt. Kashibai Navale Medical College and General Hospital, Pune (approval number: EC/NEW/INST/2022/MH/0185). The study participants were included after the submission of written informed consent. As this was a prospective observational study, clinical trial registration was not required.

Study population

A total of 60 consecutive male patients aged 40-65 years presenting to the outpatient clinic with erectile dysfunction were enrolled. Erectile dysfunction was defined using the Sexual Health Inventory for Men (SHIM) questionnaire. All participants completed the planned three-month follow-up.

Inclusion criteria

The participants were enrolled if they met the following criteria: male, aged 40-65 years, a diagnosis of erectile dysfunction based on the Sexual Health Inventory for Men (SHIM) score, and the presence of at least one comorbid condition, including diabetes mellitus, hypertension, dyslipidemia, a history of prostatic surgery, or Peyronie’s disease [9].

Exclusion criteria

We excluded the participants with any of the following: major psychiatric illness or active malignancy, contraindications to study medications, refusal to participate, or inability to comply with follow-up assessments.

Clinical assessment

Baseline evaluation included medical history, physical examination, and the assessment of erectile function using the SHIM questionnaire. Follow-up assessments were conducted monthly for three months [9].

Laboratory Investigations

Baseline and monthly investigations included the following: complete blood count (CBC), C-reactive protein (CRP), fasting blood sugar (FBS), postprandial (PP) blood sugar, glycated hemoglobin (HbA1c), liver function tests, renal function tests, lipid profile, serum testosterone (total) during morning fasting hours under standardized conditions, prostate-specific antigen (PSA), and urine routine analysis.

Penile Vascular Assessment

Penile Doppler ultrasonography was performed at baseline to evaluate arterial inflow and veno-occlusive competence [10]. Repeat Doppler assessment was not performed during follow-up.

Interventions

All participants received routine clinical management based on prevailing standard-of-care practices. The intervention reflected real-world multimodal treatment rather than a standardized experimental protocol.

Lifestyle Modification

All participants received standardized counselling and were advised to follow the following: eat a whole-food, plant-based diet; do daily aerobic exercise for 30-40 minutes; maintain sleep hygiene aimed at 6-8 hours per night; reduce alcohol intake; and abstain from smoking. Adherence to lifestyle recommendations was reviewed at each follow-up visit through patient self-report.

Pharmacological Management

For the management of ED, all the participants received tadalafil 5 mg orally once daily for three months. Adjunctive agents, including L-arginine (2.5 g/day) and fenugreek extract (1 g twice daily), were prescribed at the discretion of the treating physician.

The management of comorbid conditions was undertaken according to standard clinical guidelines and included the following: diabetes mellitus: metformin, dapagliflozin, and/or imeglimin; hypertension: losartan; and dyslipidemia: rosuvastatin. Medication selection and dose adjustments were individualized based on clinical judgment and standard treatment guidelines. No attempt was made to isolate the effects of individual medications.

Follow-Up and Monitoring

Patients were evaluated monthly for three months. Each visit included the following: SHIM scoring, a review of adherence to lifestyle modification protocol, monitoring for adverse drug effects, and repeat laboratory investigations.

Outcome measures

Primary Outcome

The primary outcome is the change in SHIM score from baseline to three months.

Secondary Outcomes

The secondary outcome is the change in HbA1c, fasting glucose, and postprandial glucose; lipid profile, particularly low-density lipoprotein (LDL) cholesterol; serum testosterone levels; and body weight and body mass index (BMI).

Statistical analysis

Continuous variables were expressed as mean ± standard deviation. Pre- and post-intervention values were compared using paired t-tests. Statistical significance for all primary and secondary outcomes was defined as a p-value of <0.05. The primary outcome measure was the change in the Sexual Health Inventory for Men (SHIM) score from baseline to three months. Secondary outcomes included changes in glycated hemoglobin (HbA1c), fasting and postprandial glucose, lipid profiles (specifically LDL cholesterol), serum testosterone levels, body weight, and body mass index (BMI). All analyses were performed using IBM SPSS Statistics version 29 (IBM Corp., Armonk, NY).

## Results

Participant characteristics

A total of 60 men aged 40-65 years (mean age: 52 ± 7 years) completed the three-month follow-up. All participants had ED and at least one associated comorbidity, including diabetes mellitus, hypertension, dyslipidemia, a history of prostatic surgery, or Peyronie’s disease. Baseline demographic and clinical characteristics are summarized in Table 1.

**Table 1:** Baseline demographic and clinical characteristics of the participants Summary of the initial demographic data and clinical status of the study population (N = 60). This table includes the mean age, types of comorbidities (e.g., diabetes and hypertension), and baseline values of the primary and secondary outcome measures, including the Sexual Health Inventory for Men (SHIM) score and metabolic markers SD, standard deviation; LDL, low-density lipoprotein; HbA1c, glycated hemoglobin

Parameters	Values
Number of participants	60
Age (years), mean ± SD	52 ± 7
Age range	40-65
Comorbidities	1-2
Duration of follow-up	3 months
Baseline SHIM score, mean ± SD	14 ± 4
Baseline testosterone (ng/dL), mean ± SD	270 ± 60
Baseline LDL cholesterol (mg/dL), mean ± SD	200 ± 50
Baseline HbA1c (%), mean ± SD	7.9 ± 1.5
Baseline body weight (kg), mean ± SD	85.0 ± 12

At baseline, the mean SHIM score was 14 ± 4, consistent with moderate ED. Mean baseline laboratory values included HbA1c of 7.9% ± 1.5%, LDL cholesterol of 200 ± 50 mg/dL, total testosterone of 270 ± 60 ng/dL, and body weight of 85.0 ± 12 kg (Table 2).

**Table 2:** Baseline laboratory parameters Mean baseline values for essential laboratory assessments prior to the start of the three-month intervention. Parameters listed include glycated hemoglobin (HbA1c), low-density lipoprotein (LDL) cholesterol, total serum testosterone, and body weight, which serve as the reference points for evaluating post-treatment changes

Parameters	Mean ± SD
HbA1c (%)	7.9 ± 1.5
LDL cholesterol (mg/dL)	200 ± 50
Total testosterone (ng/dL)	270 ± 60
Body weight (kg)	85.0 ± 12

Post-treatment outcomes

After three months of combined lifestyle and pharmacological interventions, the patients showed significant improvement across all measured domains. No severe adverse drug reactions were reported. Mild dyspepsia (n = 4) and myalgia (n = 2) were noted and resolved spontaneously.

Primary Outcome: Changes in Erectile Function

**Figure 1:**
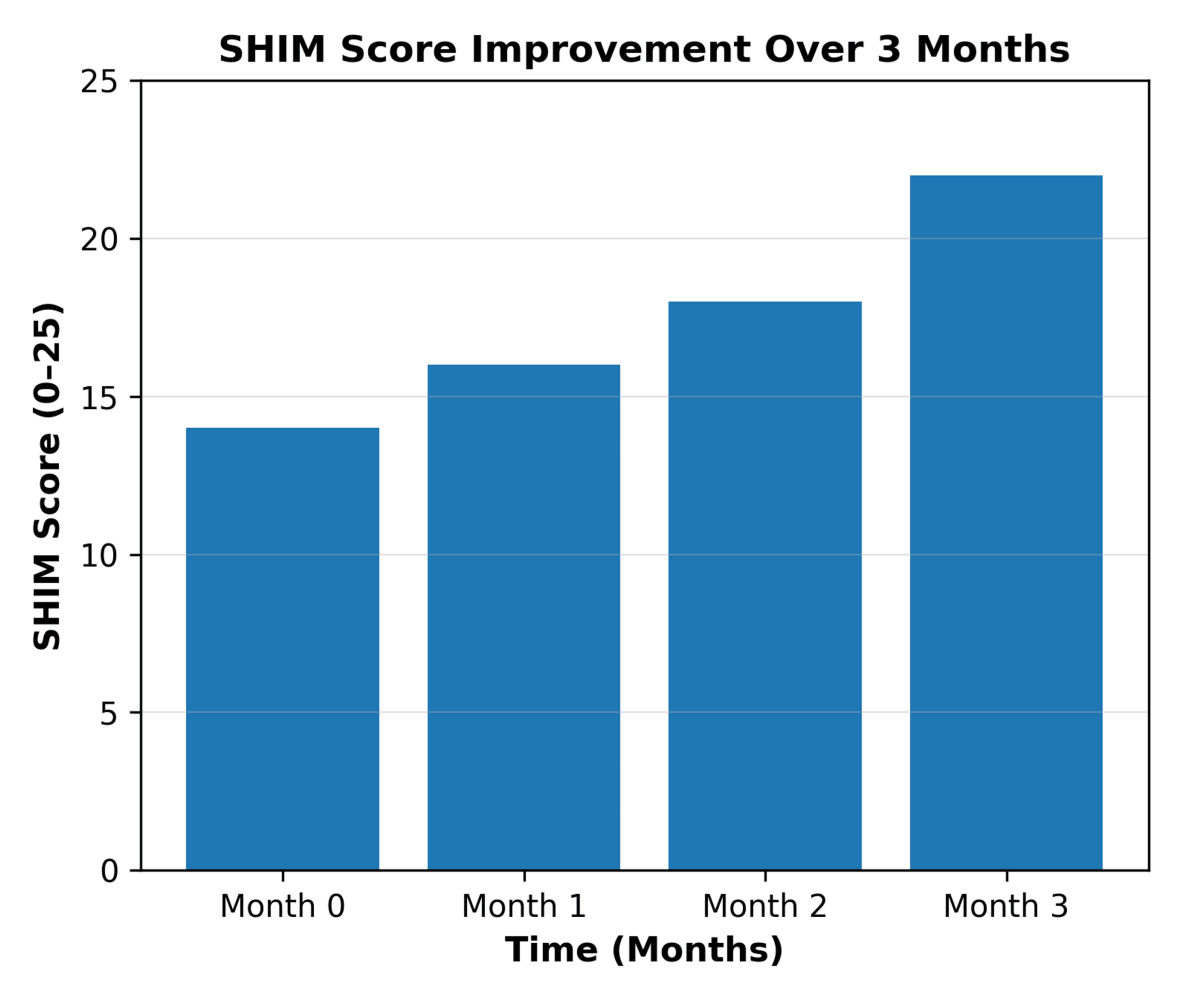
Improvement in Sexual Health Inventory for Men (SHIM) scores over three months Bar chart showing mean SHIM scores at baseline (month 0) and at monthly follow-up visits through month 3. Scores are presented on a scale of 0-25, with higher scores indicating better erectile function. Values represent mean scores at each time point

Following three months of combined lifestyle and pharmacological intervention, the mean SHIM score increased from 14 ± 4 at baseline (moderate ED) to 22 ± 5 at three months. This represented a mean increase of eight points (95% CI: 6.2-9.7). Most participants improved from moderate to mild ED, with scores approaching the normal range (22-25). This improvement is associated with enhanced sexual health and quality of life. The change in SHIM score was statistically significant (t = 8.24; p < 0.05). Monthly SHIM scores demonstrated a progressive increase over the follow-up period. This confirms the effectiveness of the combined intervention, which produced consistent and meaningful gains in erectile function among men with cardiometabolic comorbidities (Figure 1).

Secondary Outcome: Metabolic and Hormonal Changes

The study showed notable improvements in essential metabolic and hormonal parameters after three months of intervention, each carrying significant physiological and clinical implications (Table 3).

**Table 3:** Post-treatment changes at three months Comparison of mean baseline to three-month post-treatment values across all primary and secondary outcome measures. The table highlights the absolute changes in SHIM scores, metabolic indicators, and hormonal levels, along with the calculated percentage change for each parameter following the combined lifestyle and pharmacological intervention SD, standard deviation; LDL, low-density lipoprotein; HbA1c, glycated hemoglobin; SHIM, Sexual Health Inventory for Men

Parameters	Baseline (mean ± SD)	Three months (mean ± SD)	Percentage change
HbA1c (%)	7.9 ± 1.5	6.8 ± 0.8	-13.92%
LDL (mg/dL)	200 ± 50	75 ± 20	-62.5%
Testosterone (ng/dL)	270 ± 60	500 ± 120	+85.19%
SHIM score	14 ± 4	22 ± 5	+57.14%
Body weight (kg)	85.0 ± 12	79.0 ± 9	-7.06%

Mean HbA1c decreased from 7.9% ± 1.5% at baseline to 6.8% ± 0.8% at three months, corresponding to a 13.9% reduction (t = 4.21; p < 0.05). Mean LDL cholesterol decreased from 200 ± 50 mg/dL to 75 ± 20 mg/dL, representing a 62.5% reduction (t = 5.56; p < 0.05). Mean total testosterone levels increased from 270 ± 60 ng/dL to 500 ± 120 ng/dL, reflecting an 85.2% increase (t = 3.90; p < 0.05). Mean body weight decreased from 85.0 ± 12 kg to 79.0 ± 9 kg, corresponding to a 7.1% reduction (t = 3.72; p < 0.05).

Paired t-test results for all primary and secondary outcomes are presented in Table 4.

**Table 4:** Paired t-test results Statistical analysis of the changes observed between baseline and the three-month follow-up. The table presents the calculated t-values and corresponding p-values for primary outcomes (SHIM scores) and secondary outcomes (HbA1c, LDL cholesterol, testosterone, and body weight), indicating the statistical significance (p < 0.05) of the clinical improvements LDL, low-density lipoprotein; HbA1c, glycated hemoglobin; SHIM, Sexual Health Inventory for Men

Parameters	T-values	P-values
SHIM score	8.24	<0.05
HbA1c	4.21	<0.05
LDL cholesterol	5.56	<0.05
Testosterone	3.90	<0.05
Body weight	3.72	<0.05

HbA1c Decrease

**Figure 2:**
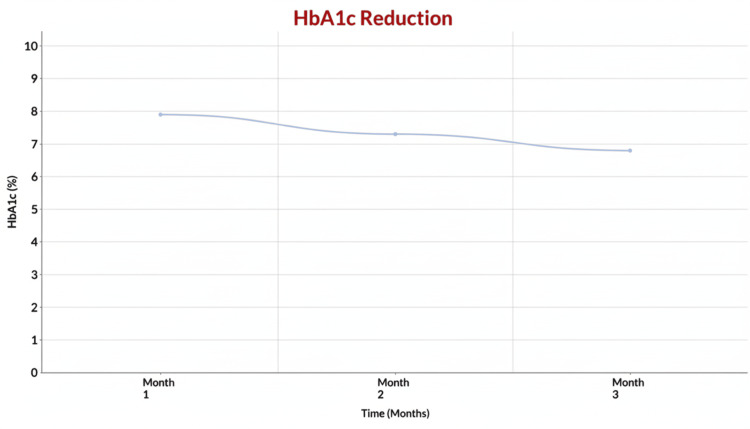
Mean HbA1c levels over the three-month follow-up period Line graph illustrating mean glycated hemoglobin (HbA1c) levels at baseline and at monthly follow-up visits over three months. HbA1c values are expressed as percentages (%). Data points represent mean values at each time point

HbA1c decreased by about 14% from 7.9% to 6.8% (t = 4.21; p < 0.05), indicating better glycemic control and a reduced risk of microvascular and macrovascular complications, which are closely associated with vascular erectile dysfunction in men with diabetes. Lower HbA1c levels are linked to improved endothelial health and erectile function over time (Figure 2).

LDL Cholesterol Reduction

**Figure 3:**
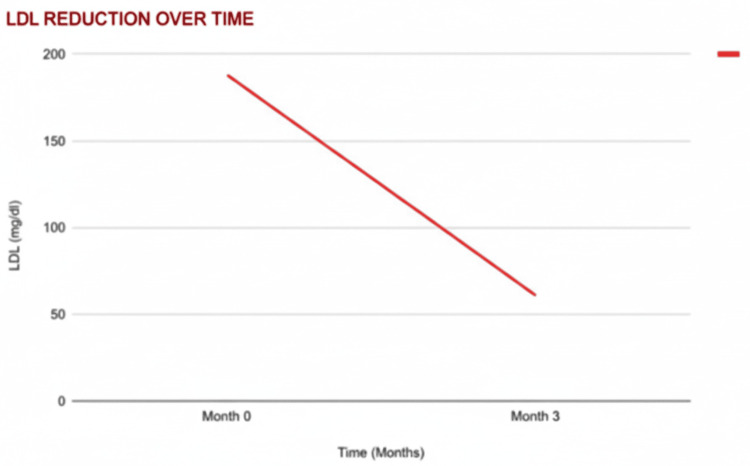
Changes in mean LDL cholesterol levels over three months Line graph depicting mean low-density lipoprotein (LDL) cholesterol levels at baseline and during the three-month follow-up period. LDL cholesterol values are expressed in milligrams per deciliter (mg/dL). Data points represent mean values at each time point

LDL cholesterol was reduced by 62.5%, decreasing from 200 mg/dL to 75 mg/dL (t = 5.56; p < 0.05), indicating improved lipid profiles (Figure 3). Lower LDL levels enhance endothelial function, increase nitric oxide availability, and support penile vascular responsiveness, all of which are significant for normal erectile function [11,12].

Testosterone Increase

**Figure 4:**
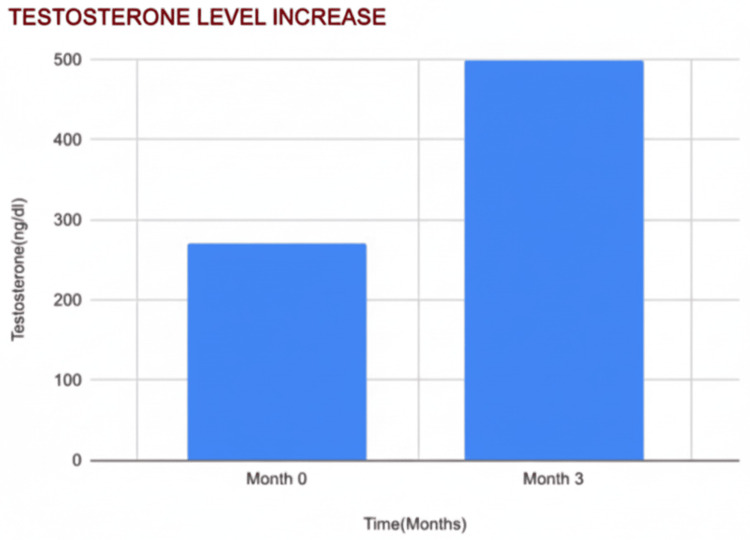
Changes in mean serum testosterone levels over three months Bar chart showing mean total serum testosterone levels at baseline (month 0) and at the three-month follow-up. Testosterone concentrations are expressed in nanograms per deciliter (ng/dL). Values represent mean measurements at each time point

Total testosterone increased by 85%, rising from 270 ng/dL to 500 ng/dL (t = 3.90; p < 0.05), signaling a reversal of obesity-related hypogonadism and enhanced metabolic health. Higher testosterone levels support nitric oxide production, libido, and erectile function (Figure 4).

Body Weight Reduction

**Figure 5:**
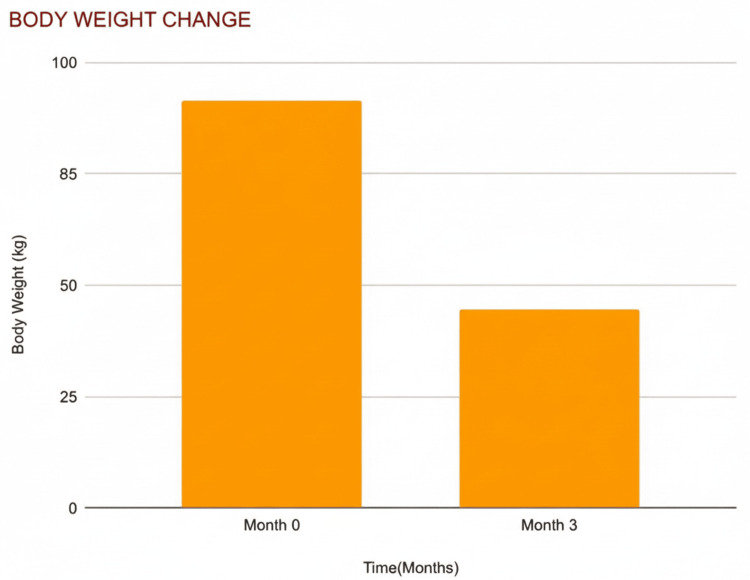
Changes in mean body weight over three months Bar chart illustrating mean body weight at baseline (month 0) and at the three-month follow-up visit. Body weight is expressed in kilograms (kg). Values represent mean measurements at each time point

Average body weight decreased by 7.1% from 85.0 kg to 79.0 kg (t = 3.72; p < 0.05), confirming effective weight loss. Even modest weight loss (≥5%) is shown to independently improve erectile function and metabolic health, especially in overweight or obese men. Weight loss also increases testosterone levels and endothelial function (Figure 5).

**Figure 6:**
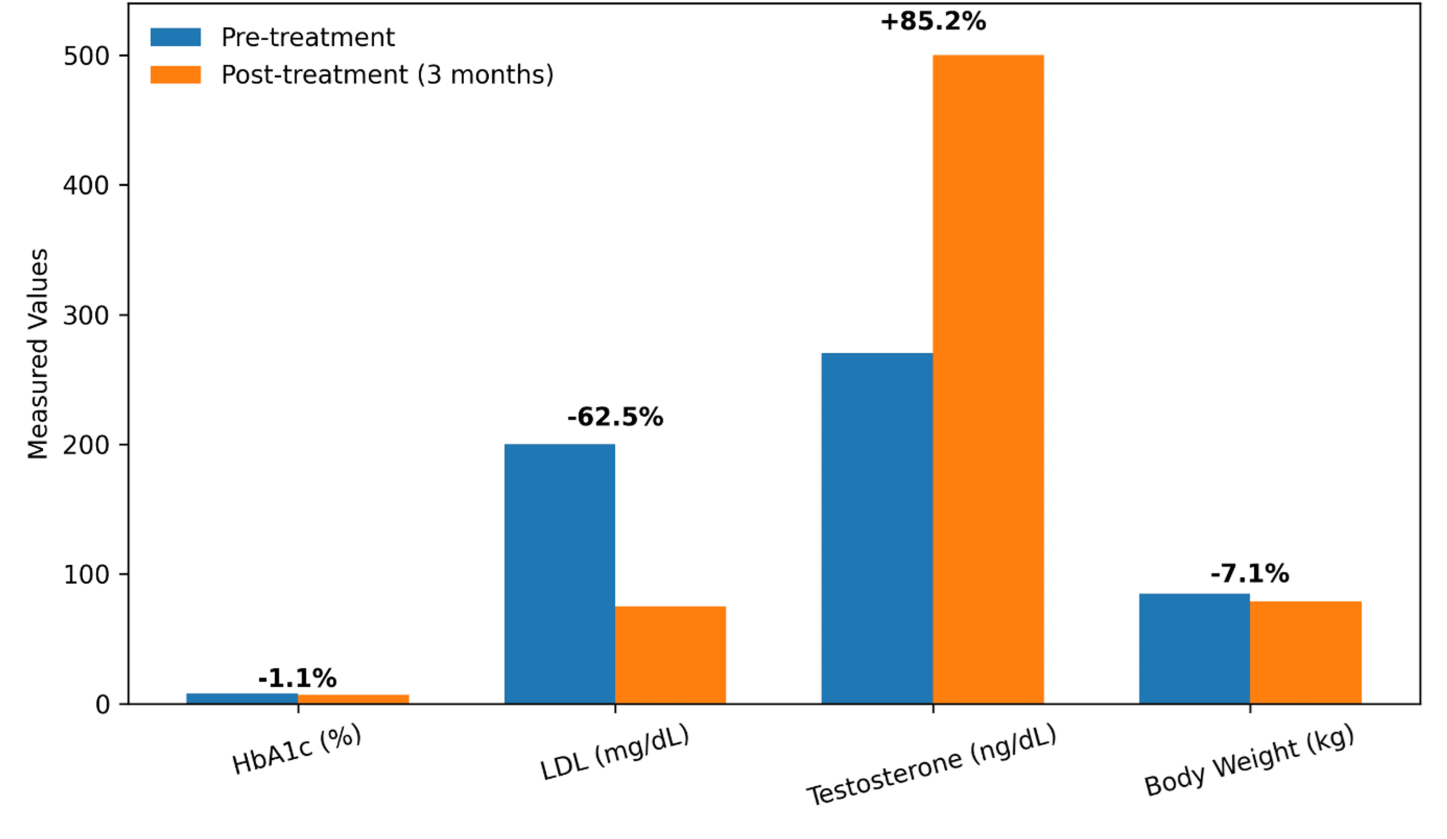
Comparison of pre-treatment to post-treatment values for metabolic and hormonal parameters Comparative bar diagram showing mean pre-treatment and post-treatment (three-month) values for HbA1c (%), LDL cholesterol (mg/dL), total testosterone (ng/dL), and body weight (kg). Percentage change from baseline is indicated above each parameter LDL, low-density lipoprotein; HbA1c, glycated hemoglobin

These results statistically support the clinical findings of the study, indicating strong effects of the intervention on erectile function, metabolism, hormonal levels, and body measurements. Paired t-tests are appropriate for comparing baseline to post-treatment measures within the same participants. Overall, these changes reinforce the effectiveness of a combined lifestyle and pharmacological approach to improving the underlying vascular, metabolic, and hormonal factors that contribute to erectile dysfunction (Figure 6).

Thus, significant reductions were observed in HbA1c, LDL cholesterol, and body weight following treatment. In contrast, a marked increase in serum testosterone levels was noted over the same period. The figure presents mean pre-treatment and post-treatment values for each parameter, with the corresponding percentage change indicated above each measure, highlighting the overall metabolic and hormonal improvements associated with the intervention.

Safety and Adverse Events

The combined therapy was well-tolerated by all participants. No serious adverse events occurred. Mild dyspepsia (n = 4) and myalgia (n = 2) were reported and resolved without intervention.

## Discussion

This prospective observational study evaluated the effects of combined lifestyle modification and pharmacological management on erectile function and selected metabolic and hormonal parameters in middle-aged men with erectile dysfunction and associated cardiometabolic comorbidities. Over a three-month follow-up period, significant improvements were observed in erectile function scores, glycemic control, lipid profile, serum testosterone levels, and body weight.

The improvement in SHIM scores from 14 ± 4 at baseline to 22 ± 5 at the end of three months suggests that a multimodal management strategy may be beneficial in men with erectile dysfunction and metabolic risk factors. ED is increasingly recognized as a manifestation of systemic vascular and metabolic dysfunction rather than an isolated genital disorder, particularly in men with diabetes, hypertension, and dyslipidemia [11-14]. Prior studies have demonstrated that phosphodiesterase type 5 inhibitors improve erectile function, but their effectiveness may be enhanced when underlying cardiometabolic abnormalities are concurrently addressed [15-18].

Improvements in glycemic control and lipid parameters observed in this cohort are clinically relevant. Specifically, mean HbA1c decreased by 13.9%, and LDL cholesterol was reduced by 62.5%. Chronic hyperglycemia and dyslipidemia are known contributors to endothelial dysfunction and impaired penile blood flow [4,14]. Better glycemic control is associated with reduced microvascular and macrovascular complications, which may indirectly influence erectile outcomes in men with diabetes [6]. Similarly, reductions in LDL cholesterol have been linked to improved endothelial responsiveness and vascular health, both of which are important determinants of erectile function [14].

A notable finding was the 85.2% increase in serum total testosterone levels over the follow-up period. Functional hypogonadism is commonly observed in men with obesity, insulin resistance, and type 2 diabetes and is often reversible with weight loss and metabolic improvement [19]. The rise in testosterone observed here may reflect broader metabolic improvements and restored hypothalamic-pituitary-gonadal axis function rather than a direct pharmacological effect. Previous studies have shown that improvements in insulin sensitivity and reductions in adiposity can restore hypothalamic-pituitary-gonadal axis function, leading to increases in endogenous testosterone levels and optimizing male reproductive outcomes [20,21]. The rise in testosterone observed in this study may therefore reflect broader metabolic improvements rather than a direct pharmacological effect.

The 7.1% reduction in mean body weight observed in this cohort further supports the role of lifestyle intervention in the management of erectile dysfunction. Excess adiposity is associated with chronic inflammation, insulin resistance, and reduced testosterone levels, all of which contribute to ED [22-24]. Evidence from randomized controlled trials and meta-analyses indicates that even modest weight loss independently improves erectile function, reinforcing weight management as an integral component of ED treatment strategies [25,26]. These findings reinforce the importance of weight management as an integral component of ED treatment strategies.

The combined therapeutic approach used in this study reflects real-world clinical practice, where men with erectile dysfunction frequently present with multiple coexisting cardiometabolic conditions. Current consensus statements and clinical guidelines recommend that men presenting with ED should undergo assessment for cardiovascular and metabolic risk factors and that lifestyle modification and risk factor optimization should accompany pharmacological treatment [27,28].

Few study results thus supported the clinical importance of integrating cardiometabolic optimization, such as blood pressure, lipid, and blood sugar control; weight loss; and exercise, along with advanced ED therapies such as vacuum erectile devices, low-frequency electrical stimulation, low-intensity extracorporeal shock waves, and Chinese acupuncture, rather than treating ED as an isolated genital issue [29]. Our study results are consistent with these recommendations and highlight the potential benefits of the integrated management of ED.

Strengths of this study include systematic follow-up, a standardized intervention protocol, and the assessment of multiple clinically relevant outcomes, including erectile function, metabolic risk markers, and hormonal parameters, thereby improving its relevance and applicability to real-world patients. However, significant limitations should be acknowledged. The absence of a control group is a considerable limitation, as it prevents attributing observed improvements to the intervention itself, a standard restriction in observational studies. The relatively small sample size and short (three-month) follow-up reduce statistical power and the ability to assess sustained benefits, a frequent limitation in trials of ED therapies. The study also did not formally assess psychological factors, even though these, including depression, performance anxiety, relationship issues, and emotional distress, are known to influence erectile function independently or synergistically with organic disease.

Our study findings suggest a positive association between the combined management approach and observed improvements in erectile and metabolic outcomes, although causal relationships cannot be established due to the observational study design. Consequently, while the study is clinically informative, further research with robust controls, larger populations, longer-term tracking, and the evaluation of psychological contributors is needed for more substantial evidence and broader clinical impact.

Future research should focus on robust randomized controlled trials with larger sample sizes and more extended follow-up periods to better determine the sustainability and generalizability of treatment effects in men with erectile dysfunction and multimorbidity. Integrating psychosocial counselling and evaluating advanced metabolic therapies, along with rigorous adherence measures to lifestyle modifications, may further improve therapeutic outcomes and clarify key mediators of success.

Limitations of the study

A control group was not used. The absence of validated psychological assessment tools (International Index of Erectile Function-Erectile Function domain {IIEF-EF}, Patient Health Questionnaire-9 {PHQ-9}, and General Anxiety Disorder-7 {GAD-7}) limits the understanding of psychogenic contributors. Penile Doppler was not repeated at three months, limiting the ability to correlate anatomical findings with functional improvement. Waist circumference was not measured and the absence of multiple comparison adjustment.

## Conclusions

An integrated management approach incorporating lifestyle modification, comorbidity optimization, and pharmacological therapy was associated with clinically meaningful improvements in SHIM scores, glycemic control, lipid parameters, body weight, and serum testosterone levels in middle-aged men with erectile dysfunction and cardiometabolic comorbidities. These findings highlight the close relationship between erectile dysfunction and cardiometabolic health and suggest that multimodal management strategies may be beneficial in routine clinical practice. However, given the observational design and the absence of a control group, the results should be interpreted as associative rather than evidence of treatment efficacy.

Future randomized controlled trials with larger sample sizes, longer follow-up, and standardized intervention protocols are needed to confirm causality, evaluate long-term sustainability, and determine the relative contributions of lifestyle and pharmacological components.

## References

[1] Rew KT, Heidelbaugh JJ (2016). Erectile dysfunction. Am Fam Physician.

[2] Ayta IA, McKinlay JB, Krane RJ (1999). The likely worldwide increase in erectile dysfunction between 1995 and 2025 and some possible policy consequences. BJU Int.

[3] Feldman HA, Goldstein I, Hatzichristou DG, Krane RJ, McKinlay JB (1994). Impotence and its medical and psychosocial correlates: results of the Massachusetts Male Aging Study. J Urol.

[4] Ray A, Ch. Maharana K, Meenakshi S, Singh S (2023). Endothelial dysfunction and its relation in different disorders: recent update. Health Sci Rev.

[5] Kaltsas A, Zikopoulos A, Dimitriadis F (2024). Oxidative stress and erectile dysfunction: pathophysiology, impacts, and potential treatments. Curr Issues Mol Biol.

[6] Defeudis G, Mazzilli R, Tenuta M (2022). Erectile dysfunction and diabetes: a melting pot of circumstances and treatments. Diabetes Metab Res Rev.

[7] Seidu S, Cebrián A, Kunutsor SK, Khunti K (2022). Erectile dysfunction, phosphodiesterase-5 inhibitor use and risk of cardiovascular disease and mortality in people with diabetes: a systematic review and meta-analysis. Prim Care Diabetes.

[8] Lu Y, Tian J, Wang S (2021). The association between plant-based diet and erectile function in Chinese young healthy men: a population-based study. Andrologia.

[9] Alwaal A, Awad M, Boggs N, Kuzbel J, Snoad B (2020). Sexual health inventory for men questionnaire as a screening method for erectile dysfunction in a general urology clinic. Sex Med.

[10] Lawal S, Igashi JB, Ibrahim MZ, Sadiku S, Musa MH, Aliyu I (2023). Doppler ultrasonographic evaluation of erectile dysfunction in a northern Nigerian tertiary hospital. J West Afr Coll Surg.

[11] Wing RR, Rosen RC, Fava JL (2010). Effects of weight loss intervention on erectile function in older men with type 2 diabetes in the Look AHEAD trial. J Sex Med.

[12] Maas R, Schwedhelm E, Albsmeier J, Böger RH (2002). The pathophysiology of erectile dysfunction related to endothelial dysfunction and mediators of vascular function. Vasc Med.

[13] Mostafaei H, Mori K, Hajebrahimi S, Abufaraj M, Karakiewicz PI, Shariat SF (2021). Association of erectile dysfunction and cardiovascular disease: an umbrella review of systematic reviews and meta-analyses. BJU Int.

[14] Wang H, Guo J, Chung E (2025). Metabolic syndrome-associated erectile dysfunction: multiple vascular endothelial dysfunction mechanisms and potential therapeutic targets. Int J Biol Sci.

[15] Maiorino MI, Bellastella G, Esposito K (2015). Lifestyle modifications and erectile dysfunction: what can be expected?. Asian J Androl.

[16] Gerbild H, Larsen CM, Graugaard C, Areskoug Josefsson K (2018). Physical activity to improve erectile function: a systematic review of intervention studies. Sex Med.

[17] Hehemann MC, Kashanian JA (2016). Can lifestyle modification affect men's erectile function?. Transl Androl Urol.

[18] Li H, Xu W, Wang T, Wang S, Liu J, Jiang H (2022). Effect of weight loss on erectile function in men with overweight or obesity: a meta-analysis of randomised controlled trials. Andrologia.

[19] Lakshmi G, Mahadevan S (2025). Diabetes and hypogonadism in males. Apollo Med.

[20] Pelusi C (2022). The effects of the new therapeutic treatments for diabetes mellitus on the male reproductive axis. Front Endocrinol (Lausanne).

[21] Kaltsas A, Dimitriadis F, Zachariou A, Sofikitis N, Chrisofos M (2025). Phosphodiesterase type 5 inhibitors in male reproduction: molecular mechanisms and clinical implications for fertility management. Cells.

[22] Moon KH, Park SY, Kim YW (2019). Obesity and erectile dysfunction: from bench to clinical implication. World J Mens Health.

[23] Ryu SY, Choi YJ, Park SY, Kim JY, Kim YD, Kim YW (2018). Udenafil, a phosphodiesterase 5 inhibitor, reduces body weight in high-fat-fed mice. World J Mens Health.

[24] Anderson SG, Hutchings DC, Woodward M (2016). Phosphodiesterase type-5 inhibitor use in type 2 diabetes is associated with a reduction in all-cause mortality. Heart.

[25] Collins CE, Jensen ME, Young MD, Callister R, Plotnikoff RC, Morgan PJ (2013). Improvement in erectile function following weight loss in obese men: the SHED-IT randomized controlled trial. Obes Res Clin Pract.

[26] Pierzak M, Głuszek S (2022). The effect of weight reduction on the sexual function and reproductive health of obese men. Stud Med.

[27] Kalka C, Keo HH, Ingwersen M (2024). Men with erectile dysfunction (ED) should be screened for cardiovascular risk factors - cost-benefit considerations in Swiss men. Vasa.

[28] Joseph JJ, Deedwania P, Acharya T (2022). Comprehensive management of cardiovascular risk factors for adults with type 2 diabetes: a scientific statement from the American Heart Association. Circulation.

[29] Pang K, Pan D, Xu H (2022). Advances in physical diagnosis and treatment of male erectile dysfunction. Front Physiol.

